# Characterization of Ku702–NLS as Bipartite Nuclear Localization Sequence for Non-Viral Gene Delivery

**DOI:** 10.1371/journal.pone.0024615

**Published:** 2012-02-08

**Authors:** Johannes Matschke, Alexander Bohla, Christof Maucksch, Rashmi Mittal, Carsten Rudolph, Joseph Rosenecker

**Affiliations:** 1 Department of Pediatrics, Ludwig-Maximilians University, Munich, Germany; 2 Institute of Animal Physiology, University of Muenster, Muenster, Germany; 3 Department of Pharmacy, Free University of Berlin, Berlin, Germany; 4 Department of Neonatology, Ludwig-Maximilians University, Munich, Germany; University of Tübingen, Germany

## Abstract

Several barriers have to be overcome in order to achieve gene expression in target cells, e.g. cellular uptake, endosomal release and translocation to the nucleus. Nuclear localization sequences (NLS) enhance gene delivery by increasing the uptake of plasmid DNA (pDNA) to the nucleus. So far, only monopartite NLS were analysed for non-viral gene delivery. In this study, we examined the characteristics of a novel bipartite NLS like construct, namely NLS Ku70. We synthesized a dimeric structure of a modified NLS from the Ku70 protein (Ku70_2_-NLS), a nuclear transport active mutant of Ku70_2_-NLS (s1Ku70_2_-NLS) and a nuclear transport deficient mutant of Ku70_2_-NLS (s2Ku70_2_). We examined the transfection efficiency of binary Ku70_2_-NLS/DNA and ternary Ku70_2_-NLS/PEI/DNA gene vector complexes *in vitro* by using standard transfection protocols as well as the magnetofection method. The application of Ku70_2_-NLS and s1Ku70_2_-NLS increased gene transfer efficiency *in vitro* and *in vivo*. This study shows for the first time that the use of bipartite NLS compounds alone or in combination with cationic polymers is a promising strategy to enhance the efficiency of non-viral gene transfer.

## Introduction

The transfer of nucleic acids into somatic cells offers new perspectives for the treatment of lethal acquired or inherited diseases. To date, effective delivery of the nucleic acids to the target cells is hindered by extracellular and intracellular biological barriers. Regarding efficiency, the most potent carrier systems are based on viral transfection systems like recombinant deficient retrovirus vectors [1], [2].

Non-viral gene delivery is limited by the low endosomal escape after cellular uptake and the low translocation of DNA into the nucleus [3], [4]. It has been shown that the endosomal escape could be improved by compacting DNA with the cationic polymer PEI which compacts and releases DNA efficiently from the endosomes into the cytosol [5]. To further improve non-viral transfection, nuclear localization sequences (NLS) have been synthesized and were used to facilitate nuclear translocation of the DNA. NLS shuttle proteins into the nucleus by binding to nuclear transport proteins such as importin α or importin β through the nuclear pore and are released in the nucleus [6]. The first studies used NLS covalently bound to pDNA. These studies proved that NLS can promote the transport of pDNA into the nucleus [7], [8], but covalently bound NLS interfered with the transgene expression of the pDNA [9]. An easier and less complicated method was developed by Ritter *et al.* by binding NLS and DNA in an electrostatic way [10].

So far, only monopartite NLS were analysed for non-viral gene delivery. In this study, we examined the characteristics of a novel bipartite NLS like construct, namely NLS Ku70, for the use as a non viral gene carrier.

## Materials and Methods

### Peptide Synthesis

Three peptides were synthesized by the department of medicine (Institute of Biochemistry, Humboldt-University, Berlin): C-KVTKRKH*GAAGAA*SKRPK-G-KVTKRKH*GAAGAA*SKRPK (Ku70_2_-NLS) as dimeric peptide of the Ku70-NLS, C-ASGSKGARPAKKRKPKRGAAHKHAGAKVRKTVTGAKK (s1Ku70_2_-NLS) as a supposed nuclear transport active mutant of the Ku70_2_-NLS and C-KTAHSKAARGHTPKGKARVVKAKAGKAKGGKAKPRSR (s2Ku70_2_) as transport deficient mutant. As far as the intervening regions of Ku70_2_-NLS are concerned the first and fourth alanine had to be replaced with glycine because 6 alanines cannot be synthesized in series. Synthesis of all peptides started with glycine. The free sulfhydryl groups of the cysteines were modified by dithiopyridin reaction in order to protect them of oxidation [11].

### Cloning of β-galactosidase fusion proteins

For subcloning of plasmid DNA coding β-galactosidase fusion proteins, we used pVAX1/lacZ plasmids (Invitrogen. UK). The coding and non-coding strand of Ku70_2_-NLS-, s1Ku70_2_-NLS and s2Ku70_2_ were synthesized by Biomers (Ulm, Germany). All annealed oligonucleotides were cloned into the pVAX1/lacZ plasmid between NheI and BamHI restriction sites. The sequencing of all cloned plasmids showed that between NLS- and β-galactosidase DNA sequence there existed one start codon and one excess nucleotide. Thereby it could not be ensured that the Ku70_2_-NLS-β-Galactosidase fusion protein could be read completely and correctly by DNA polymerase. The excess nucleotide led to a frame shift; therefore the open reading frame of β-galactosidase DNA sequence was disarranged. In order to exclude the nucleotide sequence GATG we conducted a site directed mutagenesis. Hence, we designed a forward primer (5′-TTGAATTCTGCAGATCGAAACATAGATCCCGTCGTTTTACAA-3′) and a reverse primer (5′-TTGTAAAACGACGGGATCTATGTTTCGATCTGCAGAATTCCA-3′) flanking the nucleotide sequence GATG. Using proof reading enzyme PFU II Ultra (Stratagene, CA, USA) we conducted an inverse PCR of the already cloned plasmid DNA by using the following PCR protocol: 2 min at 94°C denaturation, 18 cycles of 20 sec denaturation at 95°C, 20 sec annealing of primer at 45°C, 90 sec elongation at 68°C, and accordingly 3 min proof reading at 68°C. Afterwards, the new plasmids were digested with enzyme DpnI (Fermentas, St. Leon-Rot, Germany). Again, after transformation in *E. coli*, new plasmids were identified by gel electrophoresis using 1% agarose gel and by sequencing (GATC Biotech AG, Konstanz, Germany). Then, the plasmids pVAX1/lacZ-Ku70_2_-NLS, pVAX1/lacZ-s1Ku70_2_-NLS and pVAX1/lacZ-s2Ku70_2_ were transformated into *E. coli* strain DH10B (ElectroMAX DH10B Cells, Invitrogen, Karlsruhe, Germany), isolated and purified by using NucleoBond® EF plasmid purification kits (Macherey-Nagel, Düren, Germany).

### Plasmid DNA

The pCLuc containing firefly luciferase (a gift by Ernst Wagner, department of pharmacy, University of Munich,) and pEGFP-N1 containing enhanced green fluorescent Protein (Clontech, Palo Alto, CA, USA) were used for *in vitro* transfections. *In vivo* experiments were conducted with ccc-pCp-Luc coding for luciferase (Invitrogen, UK). For β-galactosidase experiments we used pVR1411 containing SV40-NLS (Biomers, Ulm, Germany), pVAX1/lacZ (Invitrogen, UK) containing β-galactosidase reporting gene as well as pVAX1/lacZ-Ku70_2_-NLS, pVAX1/lacZ-s1Ku70_2_-NLS and pVAX1/lacZ-s2Ku70_2_.

### Size measurement

Particle size was determined by dynamic light scattering (Brookhaven Instruments Corporation, Austria). Gene vector complexes were generated as described above in double-distilled water and PBS. Measurements were performed using the following settings: 10 sub-run measurements per sample; viscosity for water 0.89 cPa; beam mode F(Ka) ¼ 1.50 (Smoluchowsky); and temperature 25°C.

### Cell Culture

BEAS-2B cells (ATCC No. CRL-9609) and 16HBE14o^−^ cells (Prof. Dr. Dieter C. Gruenert, University of Vermont, Burlington, VT, USA), a human bronchial epithelial cell line, and HELA (DSMZ No: ACC 57, Germany), a cervical carcinoma cell line, were cultured in minimal essential medium (MEM, Gibco/Invitrogen, Karlsruhe, Germany) containing 10% fetal bovine serum (PAA Laboratories, Austria). All cells were maintained at 37°C in a 5% CO_2_ humidified air atmosphere.

### Preparation of Gene Vector Complexes

Gene vector complexes were generated in HBS (150 mM NaCl, 10 mM HEPES, pH 7.4) or PBS. For formulating binary gene vector complexes 0.5 µg DNA and a varying amount of GTA depending on the ± ratio were dissolved in 75 µl of solvent. The DNA solution was pipetted to the GTA solution and mixed vigorously by pipetting up and down. The complexes were incubated at room temperature for 20 min before use. Ternary complexes were formulated in the same way, but 0.5 µg of DNA, NLS and PEI (average molecular mass of 25 kDA; Sigma Aldrich, Deisenhofen, Germany; dialyzed against water, 12–14-kDa molecular mass cut-off and adjusted to pH 7) were diluted in 25 µl solvent per GTA. Initially, DNA solution was pipetted to the NLS solution and incubated for 10 min. Accordingly PEI was added, the ternary solution was mixed vigorously and incubated for further 10 min before use.

### *In vitro* electroporation of Ku70_2_-NLS-β-galactosidase fusion proteins

Electroporation was only used for β-galactosidase experiments and performed with BioRad GenPulser II apparatus.

### *In vitro* transfection/magnetofection and luciferase activity measurement

For transfection and magnetofection experiments, cells (10.000/well) were seeded in 96 well plates (Techno Plastic Products AG, Trasadingen, Suisse). For transfection experiments, complexes were pipetted in each well and incubated. 4 h later, the medium was replaced with 200 µl 10% FCS containing MEM supplemented with 0.1% (v/v) penicillin/streptomycin and 0.5% (v/v) gentamycin (Gibco/Invitrogen). 24 h later luciferase activity was measured after cell lysis by addition of 100 µl lysis buffer to each well (250 mM Tris, 0.1% Triton, ph = 7.8) and incubated at room temperature for 15 min. [12]. Luciferase expression was measured with Wallac Victor^2^/1420 Multilabel Counter (PerkinElmer; Rodgau-Jügesheim, Germany). The protein content was determined by a standard Bio-Rad protein assay (Bradford method). Magnetofection experiments were performed accordingly, but after having pipetted complexes to the wells, 96 well plates were placed on a sintered Nd-Fe-B magnet (NeoDelta; remanence Br, 1080–1150 mT), purchased from IBS Magnet (Berlin, Germany). Dimension of the magnet: cylindrical; d = 6 mm, h = 5 mm, inserted in an acrylic glass template in 96-well microplate format with strictly alternating polarization. Cells were incubated for 20 minutes instead of 4 h for transfection.

### FACS analysis

For FACS measurements, 100,000 cells per well were seeded in 24-well plates (TPP, Trasadingen, Switzerland). Magnetofection was performed as described above. For FACS measurements cells were washed with PBS. Then the cells were trypsinized and measured using a Becton Dickinson FACScan (San Jose, USA).

### Animal experiments and administration of gene vectors into the lung

Female BALB/c mice were purchased form Janvier (Elevage Janvier, Le Genst St. Isle, France) and maintained under specific pathogen free conditions. All experiments were approved and controlled by local ethic committee and conducted according to the guidelines of the German law of protection of animal life. Animals were anaesthetized by intraperitoneal injection of a mixture of medetomidine, midazolam, and fentanyl. The amount of plasmid DNA administered per mouse was 30 µg of CpG-free pCpGLuc. Gene transfer agents were diluted in double distilled water (Fresenius AG, Bad Homburg, Germany) in a volume of 100 µl per mouse. Firstly, branched PEI (N/P-ratio = 10) and Ku70_2_-NLS-, s1Ku70_2_-NLS and s2Ku70_2_ (+/− ratio = 5) were mixed and incubated for 10min. Afterwards, DNA solution was pipetted directly to PEI/Ku70_2_-NLS solution. Then, 100 µl of gene transfer solution was drop-wise pipetted onto the nose of each mouse. After application, mice were administered an antidote dose which consisted of atipamezol (50 µg/kg), flumazenil (10 µg/kg) and naloxon (24 µg/kg). The mice recovered from anaesthesia within 15 min and no adverse effects of the anaesthesia were observed. 24 h later, the efficiency of the gene vector application was measured by bioluminescence (IVIS 100 imaging system; Xenogen, Almeda, CA). For this purpose, mice were anaesthetized again. Signals were quantified and analyzed using the Living Image Software ver. 2.50. After imaging, anaesthetized mice were killed. Then, the posterior vena cava exit was cut and 1 ml of an isotonic sodium chloride solution was perfused slowly into the right heart in order to wash blood from the lungs and to avoid interference with the subsequent luciferase assay. The lungs were dissected from animals, frozen in liquid nitrogen and stored at −80 C. For the measurement of luciferase activity, minced lungs were each mixed with 400 µl of cell lysis buffer with addition of protease inhibitors (Roche Protease Inhibitor Cocktail Tablets). Samples were vortexed and centrifuged at 10,000 g at 4 C for 10 min. 100 µl of supernatant were measured for luciferase activity in a Lumat LB 9507 instrument (Berthold, Bad Wildbad, Germany) in duplicates by injecting 100 µl luciferin assay buffer. The emitted light was measured over 30 sec. The background was subtracted from the reported values.

### Statistical Analysis

Statistical analyses were carried out using IBM SPSS 19.0 (Chicago, IL, U.S.A.). Means and one standard deviation of a representative experiment performed in triplicates are reported. A p value<0.05 was considered to be significant. Intergroup analyses were carried out using the Mann-Whitney U test as the data were not normally distributed in all groups.

## Results

### Proof of nuclear localization activity

**Figure 1:**
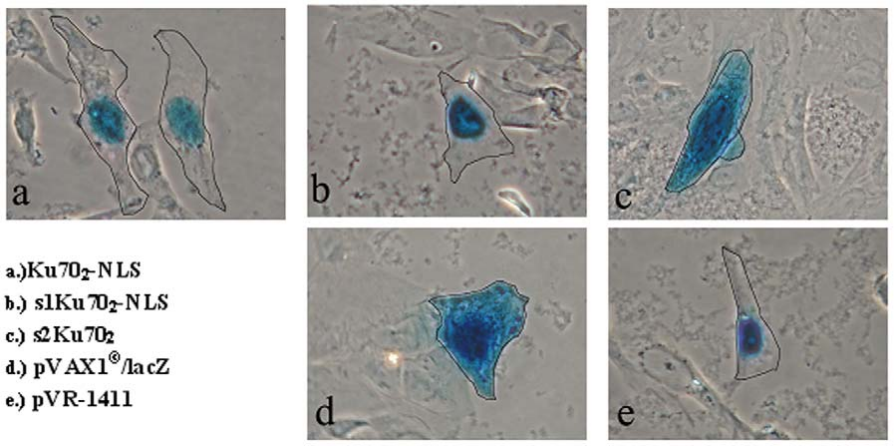
Intracellular localization of β-galactosidase fusion proteins. BEAS-2B cells were electroporated with 10 µg β-galactosidase coding plasmid DNA. All images show the typical blue β-galactosidase staining. Images (a) and (b) point that Ku70_2_-NLS and s1Ku70_2_-NLS show nuclear localization signal activity. The exclusive blue staining of nuclei is clearly visible. The s2Ku70_2_ shows staining of the cytosol. The NLS large antigen (e) was used as positive control for nuclear localisation and β-galactosidase without NLS (d) as negative control.

First, we analyzed the nuclear localization activity of the newly synthesized bipartite peptides Ku70_2_-NLS, s1Ku70_2_-NLS, and s2Ku70_2_. These examinations had to be conducted because Ku70_2_-NLS was synthesized as a dimer and the intervening region of Ku70_2_-NLS had been changed which could have influenced the nuclear localization activity. The analysed cells showed the typical blue β-galactosidase coloration after staining with β-galactosidase staining solution [13]. If the tested peptides had characteristics of a nuclear localization sequence only the nucleus should stain blue. Otherwise the complete cytosol would show the blue staining. Using this method, we could show that Ku70_2_-NLS and s1Ku70_2_-NLS had nuclear localization activity, whereas s2Ku70_2_ did not show any nuclear localization activity (Figure 1).

### Biophysical properties of binary and ternary Ku70_2_–NLS gene vector complexes

The size of particles is influenced by the solvent in such a way that ionic solvents enlarge particle size, and non-ionic solvents like distilled water result in a smaller size of the complexes [11], [14], [15], [16], [17]. Analysing the synthesized gene vector complexes for a period of 10 minutes, we could show that Ku70_2_-NLS/DNA, s1Ku70_2_-NLS/DNA and s2Ku70_2_/DNA complexes (±5) that were generated in distilled water had a constant size of about 80 nm and transMag^PEI^/DNA complexes of about 190 nm. Ternary Ku70_2_-NLS/transMag^PEI^/DNA, s1Ku70_2_-NLS/transMag^PEI^/DNA and s2Ku70_2_/transMag^PEI^/DNA are just as large as transMag^PEI^/DNA complexes and constant in size for about 10 minutes. Ternary gene vector complexes which were generated in PBS increased continuously during 20 minutes of measurement. All complexes showed a comparable size of about 300 to 400 nm.

### Comparison of the bipartite Ku70_2_–NLS with monomeric nuclear localization sequences

**Figure 2:**
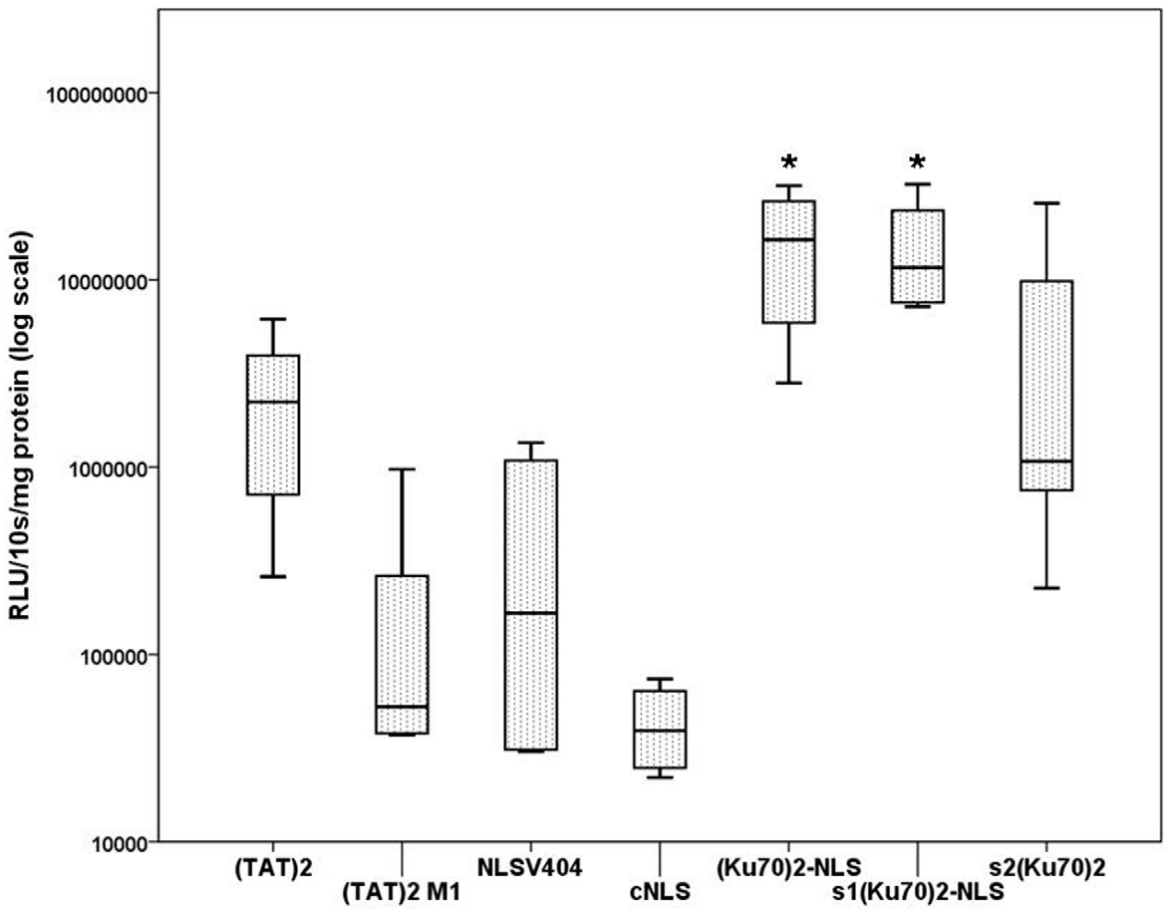
Ku70_2_-NLS in comparison to monopartite nuclear localization sequences. BEAS-2B cells were transfected with Ku70_2_-NLS/DNA complexes or the monopartite active NLS (TAT)_2_ or NLSV404, and their nuclear transport deficient sequences (control) NLS (TAT2M1 or cNLS). All the complexes were generated in HBS (charge ± = 5). Transgene expression of Ku70_2_-NLS and s1Ku70_2_-NLS was significantly higher (p<0.05, Mann-Whitney-U-Test) compared to the respective monopartite NLS (TAT)_2_ or NLSV404. Transgene expression of Ku70_2_-NLS and s1Ku70_2_-NLS compared to the control peptide s2Ku70_2_ was close to significance. This study clearly suggests an advantage of the bipartite NLS over monopartite NLS.

Ku70_2_-NLS, s1Ku70_2_-NLS were analyzed in comparison to monopartite nuclear localization sequences. We used NLSV404 (origin of SV40 virus) and TAT_2_, and their analogous nuclear transport deficient mutants cNLS and TAT_2_M1, as well as s2Ku70_2_. Gene vector complexes were generated with charge ratio of ± = 5 and transfected to BEAS-2B cells. Gene transfer efficiency mediated by Ku70_2_-NLS was significantly higher as compared to TAT_2_ or to NLS404. The same was the case for s1Ku70_2_-NLS (Figure 2). This study clearly shows an advantage of the bipartite NLS over monopartite NLS.

### Transfection efficiency depending on the +/−ratio

Plasmid DNA was complexed with Ku70_2_-NLS or with s2Ku70_2_. Transfections were performed with different charge ratios at a DNA dose of 0.5 µg. Using different +/−ratios in BEAS-2B cells and 16-HBE cells, we found that gene transfer efficiency at a ratio of ± = 5 was highest and future used.

### Ternary gene vector complexes: combination of Ku70_2_–NLS with polyethylenimine (PEI)

**Figure 3:**
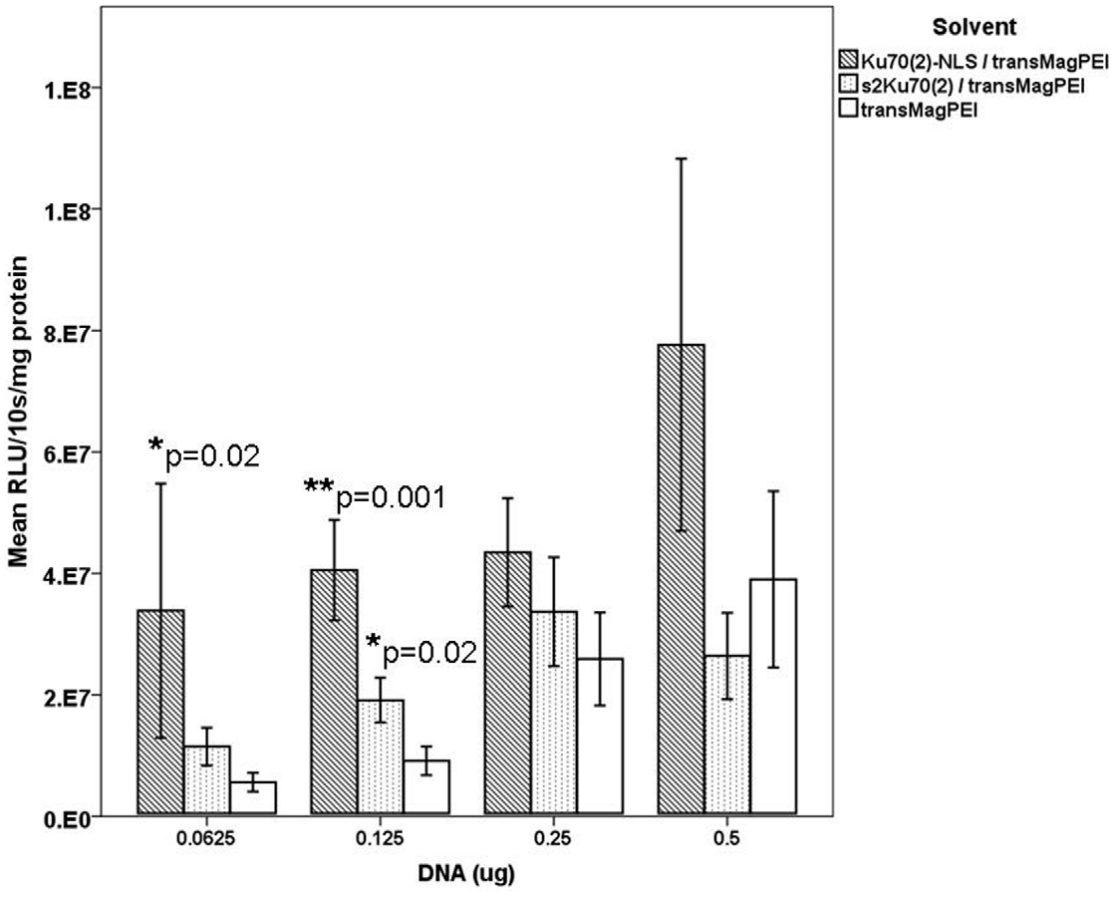
Transfection efficiency of ternary gene vector complexes using transMag^PEI^ and different solvents. BEAS-2B cells were transfected with gene vector complexes. Transgene expression of Ku70_2_-NLS/transMag^Pei^/DNA is more efficient than s2Ku70_2_/transMag^Pei^ and transMag^Pei^/DNA. *p = 0.02 and **p = 0.001.

In a first step, we formulated ternary gene vector complexes with a DNA dose of 0.25 µg at a ± ratios of 5, 2.5, 1.25, 0.625 and 0.313. As a control, binary PEI/DNA (± = 10) complexes were used. Ku70_2_-NLS/PEI/DNA and s2Ku70_2_/PEI/DNA mediated higher gene transfer efficiency than binary PEI/DNA complexes. Transgene expression mediated by Ku70_2_-NLS/PEI/DNA is about 1.7-fold to 8-fold higher as compared to transgene expression mediated by PEI/DNA. Using ± ratio of 5 Ku70_2_-NLS/PEI/DNA complexes showed the most efficient gene delivery compared to PEI/DNA. ± ratios of 2,5 and 5 tested for Ku70_2_-NLS/PEI are about 1.7-fold to 3.4-fold higher compared to s2Ku70_2_/PEI (data not shown). Furthermore, we analyzed the transfection efficiency using the magnetofection method [18]. [19]. Gene vector complexes were formulated in PBS. DNA doses 0.03125, 0.0625, 0.125, 0.25 and 0.5 µg were analyzed. Ku70_2_-NLS/transMag^Pei^/DNA complexes solvented in PBS mediated 1.7-fold to 6-fold higher transgene expression compared to transMag^Pei^/DNA. Transfection efficiency of Ku70_2_-NLS/transMag^Pei^/DNA compared to s2Ku70_2_/PEI/DNA is about 1.1-fold to 3-fold higher (Figure 3).

### Quantification of the number of transfected cells

The number of transfected cells was investigated with FACS analysis. 16HBE14o^−^ and BEAS-2B cells were transfected using magnetofection. The highest number of EGFP positive 16HBE14o^−^ cells was found in the following order: Ku70_2_-NLS/transMag^PEI^>s2Ku70_2_/transMag^PEI^>transMag^PEI^ (10.4%>8.2%>2.2%). The incremental factor of luciferase expression (Ku70_2_-NLS/transMag^PEI^: 8-fold, s2Ku70_2_/transMag^PEI^: 2.4-fold) compared to incremental factor of a number of transfected cells (Ku70_2_-NLS/transMag^PEI^: 1.5-fold, s2Ku70_2_/transMag^PEI^: 1.5-fold) clarifies that the Ku70_2_-NLS/transMag^PEI^ mediated luciferase expression is 5.3-fold (s2Ku70_2_/transMag^PEI^: 1.6-fold) compared to the number of transfected cells. In this respect Ku70_2_-NLS/transMag^PEI^ enhances gene transfer by enhancing transgene expression of each cell, but not by the number of transfected cells.

### *In vivo* application of ternary Ku70_2_-NLS/PEI/DNA and s1Ku70_2_-NLS/PEI/DNA complexes using nasal instillation

**Figure 4:**
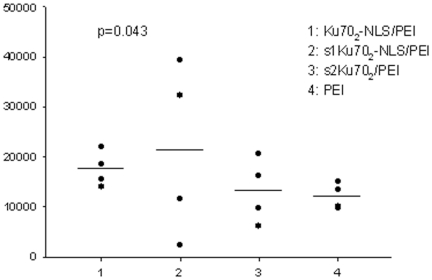
Analysing luciferase expression in lung homogenisates after nasal instillation of gene transfer agents. 1, Ku70_2_-NLS/PEI/DNA; 2, s1Ku70_2_-NLS/PEI/DNA; 3, s2Ku70_2_/PEI/DNA; 4, PEI/DNA. Gene vector complexes were formulated as follows: 30 µg DNA; NLS: ± ratio = 5; PEI: N/P ratio = 10. Luciferase activity was measured in lung homogenisates 24 h after application of gene vectors. Values between Ku70_2_-NLS/PEI and PEI significantly different (*p*≤0.043; n = 4). Statistical analysis was performed using the Mann-Whithney-U test.

Using the IVIS *in vivo* imaging method it could be established that every type of gene vector complex was able to mediate gene transfer. Luciferin luminescence was measurable over all segments of the lungs of the tested groups of mice. In some contrast to the *in vitro* data, the Ku70_2_-NLS/PEI/DNA mediated gene transfer was about 20% lower than PEI/DNA, but this effect was not statistically significant. s1Ku70_2_-NLS/PEI/DNA mediated gene transfer was about 12*%* and s2Ku70_2_/PEI/DNA mediated gene transfer about 28% higher compared to PEI/DNA (n.s.). In addition to the *in vivo* measurements with IVIS, lung homogenisates were analyzed for the presence of luciferase activity. In this analysis, the Ku70_2_-NLS/PEI mediated gene transfer was about 46% higher, s1Ku70_2_-NLS/PEI/DNA about 77% and s2Ku70_2_/DNA about 9% higher compared to PEI/DNA. The values are significantly different from control (*p*≤0.043; n = 4) (Figure 4). Although the IVIS measurements may not have reflected the in vitro results, the analyses in lung homogenisates partly confirmed the in vitro data.

## Discussion

It is known that the nuclear membrane in eukaryotic cells is a major barrier for efficient gene transfer using non-viral vectors. Based on this information we pursued a new strategy of using a bipartite nuclear localization sequence of the Ku70 protein in order to develop a more efficient non-viral gene transfer system compared to classical non viral gene transfer agents. This Ku70 protein is a subunit of the Ku protein which was found in patients with systemic lupus erythematosus and scleroderma-polymyositis overlap syndrome. This protein is involved in DNA double-strand break repair and transcription. The Ku70 subunit consists of two basic subregions and a nonbasic intervening region [20], [21]. Insofar, this NLS was interesting for our examination because the intervening region consisting of the aminoacids DNEGSG can be substituted by six alanines without any loss of NLS-functionality [21]. Substituting the negatively charged aminoacids could improve the binding intensity between NLS and pDNA. Rudolph et al. observed that the application of dimeric NLS reached the most efficient gene delivery in their study [11]. In analogy to this study, we characterized this dimeric structure of the Ku70 NLS for the use as a non viral gene carrier.

Firstly, NLS activity of the newly synthesized Ku70_2_-NLS and s1Ku70_2_-NLS was confirmed by using β-galactosidase fusionproteins. Comparing the transfection efficacy of the newly synthesized bipartite NLS with standard polyfection (PEI/DNA) we found better transfection results of Ku70_2_-NLS when using lower DNA doses (0.125 µg–0.25 µg). In contrast, PEI/DNA mediated 2.5-fold to 7-fold higher transfection efficiency in the higher dose range of DNA (0.125 µg–0.5 µg). As a result of better transfection efficiencies using lower DNA doses, the amount of DNA, peptides and PEI could be reduced considerably. Lower DNA doses lead to a reduction of toxic effects by gene transfer complexes. Therefore we used a DNA dose of 0.25 µg in the following transfections.

Using FACS analysis it was clearly visible that by transfecting Ku70_2_-NLS/transMag^Pei^/DNA complexes the number of transfected cells was higher compared to binary transMag^Pei^/DNA complexes. This result could be confirmed on both cell lines. Ku70_2_-NLS enhanced gene expression stronger by enhancing transgene expression per cell than enhancing number of transfected cells. This result was not observed after transfections with s2Ku70_2_. The better transfection results of Ku70_2_-NLS compared to s2Ku70_2_ rely on the fact that Ku70_2_-NLS is a NLS.

The results of *in vitro* transfections revealed that Ku70_2_-NLS/PEI/DNA particles are able to transfect effectively human bronchoepithelial cells. For this reason an *in vivo* application of Ku70_2_-NLS/PEI/DNA and s1Ku70_2_-NLS/PEI/DNA complexes was conducted. In these experiments an enhancement of gene transfer mediated from Ku70_2_-NLS (46%) or s1Ku70_2_-NLS (77%) compared to PEI/DNA complexes was observed.

In conclusion, ternary gene vector complexes consisting of Ku70_2_-NLS, PEI and DNA represent an effective gene delivery system. A clear enhancement of transgene expression was observed compared to PEI/DNA. Differences between Ku70_2_-NLS and s1Ku70_2_-NLS are marginal, compared to the nuclear transport-deficient s2Ku70_2_. Gene transfer efficiency using the bipartite NLS Ku70_2_-NLS improves transgene expression compared to monopartite NLS. For *in vivo* applications Ku70_2_-NLS and s1Ku70_2_-NLS have to be further optimized. Ku70_2_-NLS and s1Ku70_2_-NLS promise gene transfer agents in the field of non-viral gene delivery.
